# Aprepitant limits in vivo neuroinflammatory responses in a rhesus model of Lyme neuroborreliosis

**DOI:** 10.1186/s12974-017-0813-x

**Published:** 2017-02-15

**Authors:** Alejandra N. Martinez, Amanda R. Burmeister, Geeta Ramesh, Lara Doyle-Meyers, Ian Marriott, Mario T. Philipp

**Affiliations:** 1 Division of Bacteriology and Parasitology, Tulane National Primate Research Center, 18703 Three Rivers Rd., Covington, LA 70433 USA; 2 Department of Biology, University of North Carolina at Charlotte, 9201 University City Blvd., Charlotte, NC 28223 USA

**Keywords:** Lyme neuroborreliosis, *Borrelia burgdorferi*, Substance P, NK-1R antagonist, Neuroinflammation

## Abstract

**Background:**

Substance P (SP) is produced at high levels in the central nervous system (CNS), and its target receptor, neurokinin 1 receptor (NK-1R), is expressed by glia and leukocytes. This tachykinin functions to exacerbate inflammatory responses at peripheral sites. Moreover, SP/NK-1R interactions have recently been associated with severe neuroinflammation and neuronal damage. We have previously demonstrated that NK-1R antagonists can limit neuroinflammatory damage in a mouse model of bacterial meningitis. Furthermore, we have since shown that these agents can attenuate *Borrelia burgdorferi*-induced neuronal and glial inflammatory mediator production in non-human primate brain explants and isolated neuronal cells.

**Methods:**

In the present study, we have assessed the role played by endogenous SP/NK-1R interactions in damaging CNS inflammation in an established rhesus macaque model that faithfully reproduces the key clinical features of Lyme neuroborreliosis, using the specific NK-1R antagonist, aprepitant. We have utilized multiplex ELISA to quantify immune mediator levels in cerebrospinal fluid, and RT-PCR and immunoblot analyses to quantify cytokine and NK-1R expression, respectively, in brain cortex, dorsal root ganglia, and spinal cord tissues. In addition, we have assessed astrocyte number/activation status in brain cortical tissue by immunofluorescence staining and confocal microscopy.

**Results:**

We demonstrate that aprepitant treatment attenuates *B. burgdorferi*-induced elevations in CCL2, CXCL13, IL-17A, and IL-6 gene expression in dorsal root ganglia, spinal cord, and/or cerebrospinal fluid of rhesus macaques at 2 to 4 weeks following intrathecal infection. In addition, we demonstrate that this selective NK-1R antagonist also prevents increases in total cortical brain NK-1R expression and decreases in the expression of the astrocyte marker, glial fibrillary acidic protein, associated with *B. burgdorferi* infection.

**Conclusions:**

The ability of a centrally acting NK-1R inhibitor to attenuate *B. burgdorferi*-associated neuroinflammatory responses and sequelae raises the intriguing possibility that such FDA-approved agents could be repurposed for use as an adjunctive therapy for the treatment of bacterial CNS infections.

## Background

The neuropeptide substance P (SP) is produced at high levels within the central nervous system (CNS) and its selective receptor, the neurokinin-1 receptor (NK-1R), is expressed by resident cells such as neurons, microglia, and astrocytes, and by immune cells that can infiltrate the CNS including macrophages and lymphocytes (as reviewed in [1, 2]). In addition to its functions as a neurotransmitter in the perception of pain and its essential role in gut motility, this tachykinin is now recognized to exacerbate inflammation at peripheral sites including the skin, lung, and gastrointestinal and urogenital tracts. Indeed, this neuropeptide appears to contribute to disease pathology for some infectious agents. For example, SP increases the bronchoconstriction and damaging cardiac inflammation following infection with respiratory syncytial virus and encephalomyocarditis virus, respectively [3, 4]. Likewise, SP contributes to the severity of inflammation associated with *Trypanosoma brucei brucei* infection, and inflammation and granuloma size in a mouse model of *Taenia solium* cysticercosis [5–7].

Recently, a number of studies have identified a similar role for SP and NK-1R interactions in neuroinflammation (as discussed in [1, 2]), and our data suggest that SP exacerbates damaging inflammation elicited within the CNS in response to disparate bacterial pathogens. We determined that the absence of SP/NK-1R interactions in SP receptor-deficient mice or prophylactic pharmacological NK-1R inhibition in wild type animals significantly reduces bacteria-induced neuroinflammation and resultant CNS damage [8, 9]. NK-1R null mice and mice treated with an NK-1R antagonist showed reduced inflammatory and maintained immunosuppressive, cytokine production, as well as decreased astrogliosis, cellularity, and demyelination following intracerebral administration of the Gram-negative bacterial pathogens *Neisseria meningiditis* and *Borrelia burgdorferi*, or the Gram-positive bacterium *Streptococcus pneumoniae* [8, 9]. These rodent studies therefore indicate that SP/NK-1R interactions are essential for the progression of damaging inflammation following bacterial CNS infection.

In the present study, we have assessed the role played by endogenous SP/NK-1R interactions in damaging CNS inflammation in an established nonhuman primate (NHP) model of Lyme neuroborreliosis using the specific NK-1R antagonist, aprepitant [10]. We have previously demonstrated that this NHP model faithfully reproduces the key features of neuroborreliosis including the development of pleocytosis, as well as the classical lesions associated with leptomeningitis of the brain and spinal cord and radiculitis observed in human patients with *B. burgdorferi*-associated CNS infection [11]. We demonstrate that inhibition of SP/NK-1R interactions limits inflammatory nervous system immune responses associated with intrathecal *B. burgdorferi* administration in rhesus macaques. This ability, and the availability of centrally acting NK-1R inhibitors that are approved for clinical use, raises the intriguing possibility that targeting SP/NK-1R interactions could be useful as an adjunctive therapy for the treatment of bacterial CNS infections.

## Methods

### Spirochetal inoculum

First passage *B. burgdorferi* strain B31 clone 5A19 spirochetes, isolated from an ear biopsy of a previously infected mouse, were grown in Barbour-Stoenner-Kelly-H medium supplemented with 6% rabbit serum and antibiotics (rifampicin at 45.4 μg/mL, phosphomycin at 193 μg/ml, and amphotericin at 0.25 μg/ml; Sigma-Aldrich, St. Louis, MO) to late logarithmic phase under microaerophilic conditions. An inoculum containing 1 × 10^8^ spirochetes/ml in RPMI 1640 medium (Invitrogen, USA) was prepared as previously described [11].

### Animals

Twenty 2.5 to 5.5-year-old rhesus macaques (*Macaca mulatta*) of Chinese origin were used in this study. All protocols were approved by the Institutional Animal Care and Use Committee of the Tulane National Primate Research Center. Fifteen rhesus macaques were anesthetized and inoculated intrathecally with 1 × 10^8^ live spirochetes into the cisterna magna, whereas five rhesus macaques were left uninfected and received 1 ml of RPMI 1640 medium after removing an equivalent volume of CSF. The establishment of in vivo *B. burgdorferi* infection was confirmed by positive culture from at least necropsy tissue sample. The first set of animals were studied for 2 weeks and included two control animals (one of which was treated with aprepitant), two infected and untreated animals, and two infected animals that were treated with aprepitant. The second set of animals were studied for 4 weeks and included three control animals (one of which was treated with aprepitant), five infected and untreated animals, and four infected animals treated with aprepitant. Animals received an average dose of aprepitant (Merck & Co, Inc., Whitehouse Station, NJ) of 28 ± 6 mg/kg per day p.o. daily, and drug treatments were started 2 days before inoculation. These doses are consistent with standard veterinary regimens for the chosen drugs in NHP, and the 4-week duration of the study precluded the development of neural pathology that we have demonstrated occurs at 8 weeks following *B. burgdorferi* infection [12].

### RNA extraction and reverse transcription

Total RNA was extracted using Trizol (Thermo Fisher Scientific, Waltham, MA), and in some experiments was further purified with the RNeasy kit (Qiagen, Hilden, Germany) as previously described [13]. RNA quantity and quality were assessed using a Nanodrop ND-1000 spectrophotometer prior to generating cDNA using the high capacity RNA-to-cDNA™ Kit (Thermo Fisher Scientific) according to the manufacturer’s instructions, or the Promega M-MLV protocol (Promega, USA).

### Semi-quantitative reverse transcription-PCR and quantitative real-time PCR

Promega flexi was utilized in semi quantitative reverse transcription-PCR (RT-PCR) to assess levels of mRNA encoding NK-1R as previously described [13, 14], and quantification was performed using Bio-Rad ImageLab software and normalized to the expression of GAPDH determined in parallel RT-PCR reactions. In addition, the levels of 24 genes known to be involved in the inflammatory response and neurogenic inflammation, and two housekeeping genes, were determined by reverse transcription quantitative real-time PCR (RT-qPCR). All primers were designed to span an exon-exon junction using primer-BLAST (NCBI) and SYBR® Green qPCR assays were performed using a 7900 Real-Time PCR System (Applied Biosystems, USA) as previously described [14]. Primer specificity was assessed from the melting curves generated after 40 cycles.

### Immunoblot analysis

Homogenates from NHP frontal cortical tissue were analyzed by immunoblot analysis as we have previously described [14, 15] using a mouse monoclonal antibody directed against human NK-1R (ThermoFisher Scientific; clone ZN003). Protein bands were detected using a Bio-Rad ChemiDoc imaging system and quantification analysis was performed using ImageLab software (Bio-Rad) normalized to the expression of the housekeeping gene product β-actin.

### BioPlex cytokine protein expression analysis

Cerebrospinal fluid (CSF) samples were collected from each animal at baseline and weeks 1, 2, 3, and 4, following infection and the concentration of cytokines and chemokines present in the (CSF) was quantified using the Bio-Plex Pro™ Human Cytokine 27-plex Assay kit (Bio-Rad, Hercules, CA) following the manufacturer’s instructions. The multiplex plate was read using a Bio-Plex 200 Suspension Array Luminex System (Bio-Rad).

### Fluorescent immunohistochemical analysis

NHP frontal cortical tissue samples were fixed with 4% paraformaldehyde, mounted in optimal cutting temperature medium and flash frozen, sectioned (16 μm) at 21 °C, and subsequently stored at −80 °C. Randomly selected sections were permeabilized with either 0.1% Triton X100 and 0.2% cold-water fish gelatin in 1× phosphate buffered saline, or 1:1 methanol to acetone, for 1 h prior to immunofluorescent staining. Samples were blocked with 5% normal goat serum at room temperature and a primary fluorochrome conjugated antibody directed against glial fibrillary acidic protein (GFAP) (Abcam; clone EPR1034Y) or an unconjugated antibody against NK-1R (ThermoFisher Scientific; clone ZN003) was added overnight at 4 °C. A fluorochrome conjugated secondary antibody to detect anti-NK-1R staining was incubated at room temperature for 1 h and coverslips were mounted with Prolong Gold with DAPI. Samples were analyzed using an Olympus IX70 Fluoview 1000 confocal microscope, and multiple images were captured in five random fields for each section. CellProfiler was utilized to quantify the mean fluorescent intensity for confocal images. Data is shown as mean fluorescent intensity normalized to the number of cells [16].

### Statistical analysis

Data is presented as the mean ± standard deviation (SD). Statistical analyses were performed using one-way analysis of variance (ANOVA) with Bonferroni’s or Tukey’s post hoc tests as appropriate using commercially available software (GraphPad Prism, GraphPad Software, La Jolla, CA). In all experiments, results were considered statistically significant when a *P* value of less than 0.05 was obtained.

## Results

### Cortical brain NK-1R expression increases in a SP/NK-1R interaction-dependent manner in a non-human primate model of Lyme neuroborreliosis

**Fig. 1:**
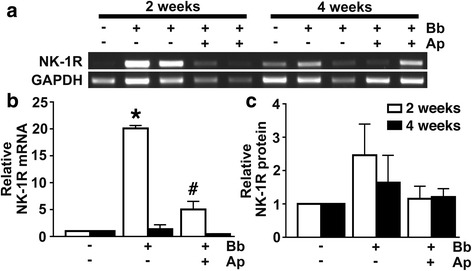
In vivo NHP infection with *B. burgdorferi* increases NK-1R expression in the CNS, and such increases are prevented by treatment with the NK-1R antagonist aprepitant. Rhesus macaques were uninfected (*n =* 2 animals) or infected intrathecally with *B. burgdorferi* (Bb, 1 × 10^8^ bacteria; *n =* 8), and infected animals were either untreated (*n =* 4) or treated with aprepitant (*n =* 4) for 2 or 4 weeks prior to euthanasia. Expression of mRNA encoding NK-1R in frontal cortical tissue samples was determined by RT-PCR (**a**) and relative expression normalized to GAPDH levels was determined by densitometric analysis (**b**). NK-1R protein expression was determined in tissue samples by immunoblot analysis and normalized to β-actin expression (**c**). Data is expressed as the mean ± SD. Asterisk and pound symbols indicate statistically significant difference from uninfected animals and untreated infected animals, respectively (*p* < 0.05)

To begin to determine the role of SP/NK-1R interactions in neuroinflammation associated with *B. burgdorferi* infection of the CNS in NHPs, we assessed NK-1R expression levels in the brain cortex of rhesus macaques at rest and following intrathecal *B. burgdorferi* infection (1 × 10^8^ bacteria). As shown in Fig. 1, expression of mRNA encoding NK-1R was significantly increased in the brain cortex at 2 weeks following infection and an elevation in NK-1R protein expression was observed although this effect failed to reach statistical significance. The effect of *B. burgdorferi* on NK-1R mRNA expression was reversed by 4 weeks following infection (Fig. 1a, b). Interestingly, the increases in NK-1R mRNA expression and the tendency to increase NK-1R protein levels at 2 weeks following infection, were not seen in animals that received treatment with the NK-1R-specific antagonist aprepitant (125 mg daily p.o) (Fig. 1).

### *B. burgdorferi*-induced increases in CSF levels of CCL2 in infected NHPs are attenuated by treatment with an NK-1R antagonist

**Fig. 2:**
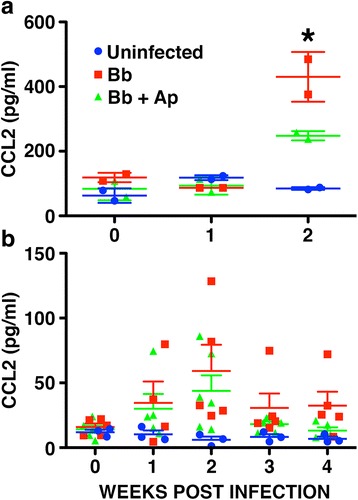
Aprepitant treatment prevents *B. burgdorferi*-induced increases in CCL2 protein levels in the CSF of NHPs. Rhesus macaques were uninfected (*n =* 5 animals) or infected intrathecally with *B. burgdorferi* (Bb, 1 × 10^8^ bacteria; *n =* 15) and were untreated (*n =* 7) or treated with aprepitant (Ap, *n =* 8) for 2 (**a**) or 4 (**b**) weeks. Expression of CCL2 in CSF was determined by multiplex analysis. Data is expressed as the mean ± SD and asterisks indicate statistically significant differences between the untreated and treated groups (*p* < 0.05)

Multiplex cytokine assay analysis showed that levels of the inflammatory chemokines CCL2 and CXCL8, and the cytokine IL-6 were significantly elevated in the CSF of infected rhesus macaques at 2 weeks following intrathecal *B. burgdorferi* infection (Fig. 2a and data not shown). Importantly, treatment with aprepitant significantly attenuated increases in CCL2 protein levels at 2 weeks following infection in this experimental series (Fig. 2a).

However, this result was not longitudinally reproducible in CSF samples from a second experimental series followed over 4 weeks post-infection as these animals showed a lower average inflammatory response to infection (Fig. 2b and data not shown). We surmised that this might be due to the confounding influence of multiple CNS regions on CSF cytokine concentrations obscuring aprepitant treatment effects. Therefore, we subsequently evaluated cytokine transcription in specific regions of the CNS that demonstrated a high number of inflammatory lesions per unit area as assessed by a pathologist. These regions included the dura mater and spinal cord, and dorsal root ganglia (DRG).

### *B. burgdorferi*-induced increases in CXCL13 and CCL2 mRNA expression in the DRG of infected NHPs are attenuated by NK-1R antagonist treatment

**Fig. 3:**
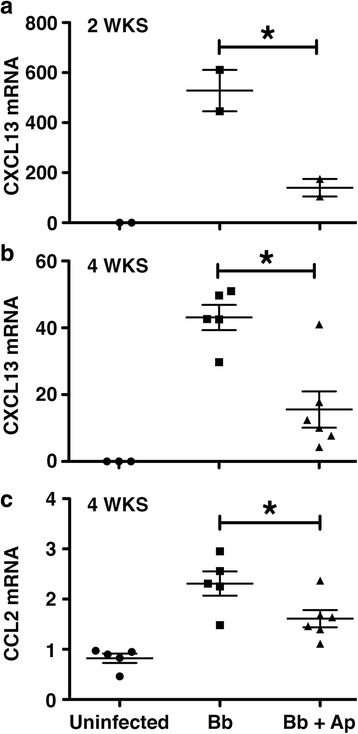
Aprepitant treatment prevents *B. burgdorferi*-induced increases in CCL2 and CXCL13 mRNA expression in the dorsal root ganglia of NHPs. Rhesus macaques were uninfected (*n =* 5 animals) or infected intrathecally with *B. burgdorferi* (Bb, 1 × 10^8^ bacteria; *n =* 15), and were untreated (*n =* 7) or treated with aprepitant (Ap, *n =* 8) for 2 (**a**) or 4 (**b**, **c**) weeks. The level of expression of mRNA encoding CXCL13 (**a**, **b**) and CCL2 (**c**) in the dorsal root ganglia was determined by qPCR. Data is expressed as the mean ± SD and asterisks indicate statistically significant differences between the untreated and treated groups (*p* < 0.05)

Analysis of mRNA expression in pooled DRG revealed that expression of mRNA encoding CXCL13 was significantly elevated at 2 and 4 weeks following *B. burgdorferi* administration (Fig. 3a, b), while levels of CCL2 mRNA were higher at 4 weeks following infection (Fig. 3c). Importantly, daily treatment with aprepitant significantly attenuated infection-associated increases in CXCL13 and CCL2 mRNA expression (Fig. 3).

### *B. burgdorferi*-induced increases in inflammatory cytokine and chemokine mRNA expression in the dura mater and spinal cord of infected NHPs are attenuated by NK-1R antagonist treatment

**Fig. 4:**
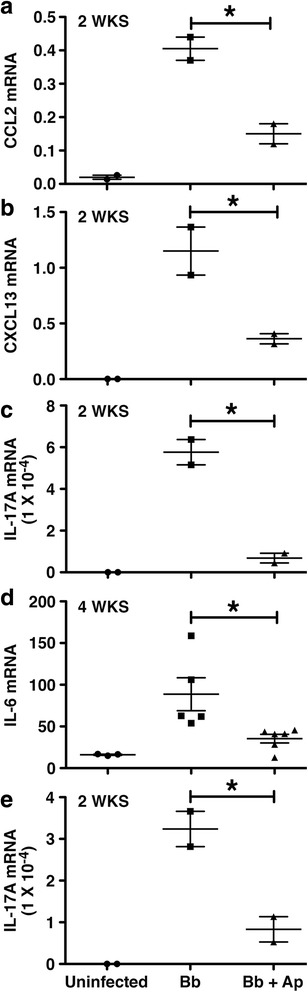
Aprepitant treatment prevents *B. burgdorferi*-induced increases in CCL2, CXCL13, IL-17A, and IL-6 mRNA expression in the spinal cord of NHPs. Rhesus macaques were uninfected (*n =* 5 animals) or infected intrathecally with *B. burgdorferi* (Bb, 1 × 10^8^ bacteria; *n =* 15) and were untreated (*n =* 7) or treated with aprepitant (Ap, *n =* 8) for 2 (**a**–**c**, **e**) or 4 (**d**) weeks. The level of expression of mRNA encoding CCL2 (**a**), CXCL13 (**b**), IL-17A (**c**, **e**), and IL-6 (**d**) in the spinal cord cervical (**a**–**d**) and thoracic (**e**) regions was determined by qPCR. Data is expressed as the mean ± SD and asterisks indicate statistically significant differences between the untreated and treated infected animal groups (*p* < 0.05)

Analysis of mRNA expression in the dura mater and spinal cord within the cervical region revealed that expression of mRNA encoding CXCL13, CCL2, and IL-17A was significantly elevated at 2 weeks following *B. burgdorferi* administration (Fig. 4a–c), while levels of mRNA encoding IL-6 were higher at 4 weeks following infection (Fig. 4d). Similarly, levels of mRNA encoding IL-17A were higher in thoracic region dura mater and spinal cord at 2 weeks following *B. burgdorferi* challenge (Fig. 4e). Importantly, daily treatment with aprepitant significantly attenuated these infection-associated increases in inflammatory mediator mRNA expression (Fig. 4).

### *B. burgdorferi*-induced decreases in cortical astrocyte marker expression are attenuated by NK-1R antagonist treatment

**Fig. 5:**
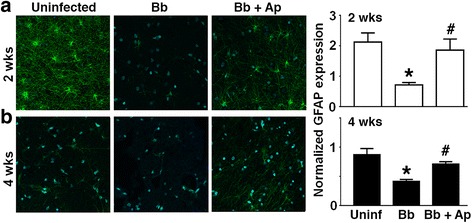
Aprepitant treatment attenuates *B. burgdorferi* infection-induced reductions in astrocyte activity/numbers. Rhesus macaques were uninfected orx infected intrathecally with *B. burgdorferi* (Bb, 1 × 10^8^ bacteria) and were untreated or treated with aprepitant (Ap) for 2 or 4 weeks prior to euthanasia. GFAP expression in frontal cortical tissue samples at 2 (**a**) and 4 (**b**) weeks following infection was determined by immunofluorescence microscopy. Relative GFAP expression in two fields of three sections from an animal in each group is shown and data is expressed as the mean ± SD. Asterisk and pound symbols indicate statistically significant difference from uninfected animals and untreated infected animals, respectively (*p* < 0.05)

To further assess the role of SP/NK-1R interactions in neuroinflammation in NHPs, we assessed the relative expression of the astrocyte marker GFAP in the brain cortex of uninfected animals and following *B. burgdorferi* infection. Immunofluorescent staining of NHP frontal cortex tissue demonstrated that levels of GFAP expression are decreased at 2 and 4 weeks following *B. burgdorferi* administration (Fig. 5). Interestingly, infection-associated decreases in GFAP expression were significantly attenuated in animals that received the NK-1R antagonist aprepitant.

## Discussion

Bacterial infections of the CNS constitute a group of highly damaging and often life-threatening diseases. What makes the etiology of these diseases so perplexing is that severe CNS inflammation can be initiated by bacterial species that are generally regarded to be of low virulence [17]. While such responses may be protective, inflammation elicited by infectious agents often results in progressive CNS damage. Indeed, we have recently demonstrated that inflammation plays a key role in pathogenesis in a NHP model of acute Lyme neuroborreliosis [12]. A hallmark of developing inflammation is the synergistic interaction between cells and their products that can amplify the response. It is now widely accepted that SP, the most abundant tachykinin in the CNS, can exacerbate the inflammatory responses of both leukocytes and resident glial cells via the high affinity NK-1R (as reviewed in [1, 2]). Importantly, we have demonstrated that SP can augment proinflammatory mediator production by murine glia in response to *B. burgdorferi* [9]. Consistent with this finding, we have shown that endogenous SP/NK-1R interactions are required for maximal proinflammatory cytokine expression in vivo following direct CNS administration of this spirochete in mice [9]. More recently, we have shown that an NK-1R antagonist can attenuate the neuronal and glial production of inflammatory mediators including CCL2 and IL-6 in rhesus macaque frontal cortex explants and isolated DRG cells following *B. burgdorferi* challenge [18].

In the present study, we have confirmed that levels of gene expression of select inflammatory cytokines and chemokines, including CCL2, CXCL13, IL-17A, and IL-6, are increased in the DRG and spinal cord tissue samples and the CSF from rhesus macaques at 2 to 4 weeks following intrathecal *B. burgdorferi* administration. An increase in IL-17A gene expression following infection is particularly interesting since cell signaling mediated by this cytokine plays a key role in regulating the expression of other inflammatory mediators, such as IL-6, via a mechanism that involves NF-κB-mediated transcription [19]. Consistent with our previous studies using frontal cortex explants and isolated DRG cells [12], treatment with the NK-1R antagonist, aprepitant, was able to significantly attenuate the transcription of inflammatory mediators in our in vivo NHP model of Lyme neuroborreliosis. Taken together, these data support the contention that endogenous SP/NK-1R interactions play a significant role in the initiation and/or progression of neuroinflammation associated with *B. burgdorferi* infection of the CNS.

Interestingly, our studies also demonstrate that NK-1R expression is increased in the NHP brain cortex at 2 weeks following infection and that this effect can be abolished by treatment with aprepitant. While the mechanisms underlying the ability of *B. burgdorferi* to increase NK-1R expression are not clear, this finding is in agreement with previous studies demonstrating the ability of bacteria and/or their products to upregulate NK-1R expression by leukocytes [20, 21]. However, the ability of aprepitant to prevent increases in NK-1R expression suggests that *B. burgdorferi*-induced effects occur secondary to a response that is, at least in part, dependent upon SP/NK-1R interactions. This would be consistent with the documented ability of inflammatory mediators to increase NK-1R expression by leukocytes [20, 21] and glial cells [22].

Finally, our immunohistochemistry analysis of NHP frontal cortex tissue demonstrates that the number and/or activation level of astrocytes as determined by GFAP expression is decreased in NHPs at 2 and 4 weeks following *B. burgdorferi* administration. While the mechanisms underlying this effect are unclear, decreases in astrocyte number/activation may result from increased apoptotic death of this population following infection or could occur as a result of the compensatory production of suppressive mediators, such as IL-10 and IL-19, that have been shown to be produced in a delayed manner by *B. burgdorferi*- challenged microglia and/or astrocytes [14, 23]. Interestingly, treatment with aprepitant prevented *B. burgdorferi*-induced decreases in GFAP expression indicating that this effect, either directly or indirectly, is mediated by endogenous SP/NK-1R interactions.

Non-peptide NK-1 receptor antagonists are known to exert central effects [24]. Of the latest generation of NK-1R antagonists, aprepitant has been shown to cross the blood-brain barrier after oral administration using human positron emission tomography to demonstrate its ability to occupy NK-1R within the brain in an oral dose and plasma concentration-dependent manner [25]. The ability of NK-1R antagonists to cross the blood-brain barrier means that these agents have the potential for use in the treatment of a wide range of CNS disorders [26–29]. Inhibitors of this tachykinin receptor have been the subject of extensive study for the clinical treatment of depression and anxiety, and aprepitant and its pro-drug fosaprepitant are currently employed clinically as post-chemotherapy anti-emetic agents [10]. While we did not formally investigate the safety of aprepitant in this study, no adverse events were recorded for any of the animals receiving this drug that could be attributed to the treatment at any point in the study period. This agrees with other studies in which aprepitant was used (performed both in humans and in mice), where no safety issues were detected [30, 31].

Given a potential role for SP/NK-1R interactions in damaging inflammatory responses within the CNS following infection, there has been considerable interest in targeting this receptor to limit neuroinflammation and neurological sequelae associated with infectious agents. For example, an NK-1R antagonist has been shown to prevent seizure activity in a rodent model of helminth brain infection [32], while our own studies have shown that pharmacological targeting of NK-1R with the antagonist L703,606 can not only prevent the development of damaging inflammation due to streptococcal CNS infection when administered prophylactically but can also reverse infection-associated gliosis and demyelination when delivered therapeutically without increasing CNS bacterial burden [8]. Furthermore, aprepitant has been tested as an adjunctive therapy for the treatment of HIV-associated neurocognitive disorder in human clinical trials and has been shown to reduce in vivo serum levels of several pro-inflammatory cytokines and chemokines, and lower CD4-positive T cell expression of sCD163 and PD-1 [33, 34]. As such, while further studies are needed to define the specific mechanisms underlying the ability of SP to augment CNS inflammation and its role in pathogen clearance, the available data raise the intriguing possibility that currently approved NK-1R antagonists, such as aprepitant, could be repurposed for use as a co-therapy to limit the neuroinflammatory damage associated with infectious agents. Clearly, further investigation of the prophylactic and therapeutic benefits of NK-1R antagonists in such conditions is warranted.

## Conclusions

Our results indicate that NK-1R antagonist treatment can attenuate aspects of bacterially induced inflammatory responses in CNS tissues in an in vivo NHP model of Lyme neuroborreliosis. Thus, in addition to antibiotics, treatment with clinically approved NK-1R antagonists could be explored as an adjuvant intervention against neuroinflammatory damage associated with infectious agents.

## Abbreviations

ANOVA: Analysis of variance
cDNA: Complementary deoxyribonucleic acid
CNS: Central nervous system
DAPI: 4′,6-Diamidino-2-phenylindole
DRG: Dorsal root ganglia
ELISA: Enzyme linked immunosorbent assay
FDA: Food and Drug Administration
GFAP: Glial fibrillary acidic protein
IL: Interleukin
mRNA: Messenger ribonucleic acid
NF-kB: Nuclear factor kappa-light-chain-enhancer of activated B cells
NHP: Non-human primate
NK-1R: Neurokinin-1 receptor
p.o.: Per os
RNA: Ribonucleic acid
RT-PCR: Reverse transcribed polymerase chain reaction
RT-qPCR: Reverse transcribed quantitative polymerase chain reaction
SD: Standard deviation
SP: Substance P
